# Ge/Si multilayer epitaxy and removal of dislocations from Ge-nanosheet-channel MOSFETs

**DOI:** 10.1038/s41598-021-04514-y

**Published:** 2022-01-19

**Authors:** Chun-Lin Chu, Jen-Yi Chang, Po-Yen Chen, Po-Yu Wang, Shu-Han Hsu, Dean Chou

**Affiliations:** 1 Taiwan Semiconductor Research Institute, NARL, Hsinchu, Taiwan; 2 General Education Centre, Tainan University of Technology, Tainan, Taiwan; 3 Department of Common and Graduate Studies, Sirindhorn International Institute of Technology, Pathum Thani, Thailand; 4 Department of Biomedical Engineering, National Cheng Kung University, 1 University Road, Tainan, 701 Taiwan; 5 Medical Device Innovation Center, National Cheng Kung University, 1 University Road, Tainan, 701 Taiwan

**Keywords:** Electronic devices, Structural materials

## Abstract

Horizontally stacked pure-Ge-nanosheet gate-all-around field-effect transistors (GAA FETs) were developed in this study. Large lattice mismatch Ge/Si multilayers were intentionally grown as the starting material rather than Ge/GeSi multilayers to acquire the benefits of the considerable difference in material properties of Ge and Si for realising selective etching. Flat Ge/Si multilayers were grown at a low temperature to preclude island growth. The shape of Ge nanosheets was almost retained after etching owing to the excellent selectivity. Additionally, dislocations were observed in suspended Ge nanosheets because of the absence of a Ge/Si interface and the disappearance of the dislocation-line tension force owing to the elongation of misfit dislocation at the interface. Forming gas annealing of the suspended Ge nanosheets resulted in a significant increase in the glide force compared to the dislocation-line tension force; the dislocations were easily removed because of this condition and the small size of the nanosheets. Based on this structure, a new mechanism of dislocation removal from suspended Ge nanosheet structures by annealing was described, which resulted in the structures exhibiting excellent gate control and electrical properties.

## Introduction

Microcircuits are ubiquitous in the modern world owing to their applicability in several fields. These complex systems consist of dozens of simple but extraordinary devices known as transistors. The ever-changing nature of technology has necessitated improvements in semiconductor efficacy to facilitate their applications in various areas. Therefore, different processing methods have been recently developed to improve the performance and boost the density of transistors, which consequently improves the performance of microprocessors. The development of fin field-effect transistors (FinFETs) by Xuejue et al.^1^ was revolutionary in this regard. This method overcame the difficulty involving the size reduction of traditional planar transistors. Efforts that have been made since then to reduce the size of transistors further have plateaued. However, the development of gate-all-around field-effect transistors (GAAFETs) has enabled the nanometre-level processing of transistors. Recently, various novel structures have been derived based on GAAFETs, such as GAAFETs with a stack of lateral nanowires and horizontally stacked nanosheet GAAFETs. However, these new structures are challenging to manufacture and have issues regarding the elimination of crystal defects. Therefore, the appropriate selection of materials and removal of crystal defects is vital. The type of material selected is known to affect transistor efficiency due to electron mobility. For decades, silicon materials have been extensively used in epitaxial wafer layers; however, they encounter physical limitations and cause a decrease in the minification efficiency after entering the 10-nm node. Therefore, major semiconductor manufacturers have worked on developing alternative materials with more excellent stability and efficiency. Among them, germanium and III–V compounds are known to effectively improve the electron mobility of transistor channels, increase chip effectiveness, and enhance power-saving benefits. Consequently, they have become the premier options for use in new semiconductor manufacturing methods. Crystal defects, on the other hand, are known to affect the water quality and must therefore be eliminated. These defects not only cause material deterioration of the epitaxial layer but can also readily form electron–hole recombination centres in the active layer, which can seriously affect the operating performance of the components.

Consequently, it is essential to employ substrates and epitaxial materials with mismatched lattice constants. The lattice mismatch between the epitaxial materials and substrates assists in the release of cumulative stress through dislocations and surface roughness during epitaxy. Previous studies by Frank et al.^2–4^ and Matthews et al.^5–7^ have described a relationship between the lattice mismatch and the thickness of the epitaxial layer. For a given lattice mismatch factor (*m*) of an epitaxial layer/substrate structure, a specific thickness known as the critical thickness (*t*_c_) exists and is defined as the following:

$$ t_{c} = \frac{{b\left( {1 - \nu cos^{2} \theta } \right)}}{{8\pi \left( {1 + \nu } \right)mcos\lambda }}\left( {ln\frac{{t_{c} }}{b} + 1} \right) $$

where ν is the Poisson ratio, b is the Burgers vector, θ is the angle between the dislocation line and its Burgers vector, and λ is the angle between the direction of slip and the direction of the film plane that is perpendicular to the line of intersection between the slip plane and the interface. Furthermore, the dislocation theory suggests that the dislocation line cannot end within the lattice; it must either form a dislocation loop or extend to the grain boundary. Therefore, the misfit dislocation generated on the heterostructure junction, which extends from the junction to the interface, increases as the thickness of the epitaxial layer increases. This extended dislocation that penetrates the interface is denoted as a threading dislocation. These defects cause the degradation of components; therefore, reducing the number of dislocations is necessary for the semiconducting process. Currently, single and stacked GAA nanowire/nanosheet structures are attracting attention for developing transistors at sub-5-nm technology nodes^8,9^. GAA structures offer excellent electrostatic and short-channel control, and the stacking of nanowires/nanosheets increases the total drive current per footprint. Si, SiGe, GeSn, and InGaAs stacked-channel FETs have been reported in the literature^10–16^.

## Experimental

Si-on-insulator (SOI) wafers with a 70-nm-thick Si top layer (p-type, 9–18 Ω∙cm) were employed as the starting substrates. The wafers were cleaned using the RCA standard cleaning 1 and 2 methods (SC1 and SC2) for removing organic materials, certain metals, and particles from the Si substrates; the wafers were subsequently rinsed in deionised water and dried in N_2_. To prepare the starting material, three periods of Ge(40 nm)/Si(25 nm) epitaxial layers were grown on SOI(100) with a 40-nm Si layer using a low-pressure chemical vapour deposition (LPCVD) system with GeH4 and SiH4 gases. The Ge/Si epitaxial multilayers were used as the starting material instead of conventional Ge/Si layers owing to better etching selectivity between Ge and Si in our etching process; this will be discussed in detail later. However, the large lattice mismatch between Ge and Si prevents Ge/Si epitaxial layers from growing in a 2D model. Therefore, the temperatures during epitaxy of Ge and Si were maintained at 400 °C and under 500 °C, respectively, to avoid island growth. The thickness of the deposited Ge film was determined by transmission electron microscopy (TEM), and no dislocations were detected in the Ge nanosheets during TEM observations conducted in parallel with photoluminescence (PL) analysis. The crystallisation of the Ge film was examined by X-ray diffraction (XRD; Cu Kα, λ = 1.5408 Å). All etchings were performed in Lam Research reactors (TCP 9600), which are transformer-coupled plasma (TCP) reactors that facilitate separate control of the coil (top electrode) power and substrate (lower electrode) bias. Backside cooling using He was carried out to enable more effective management of the substrate temperature. Samples were mounted on a 6-in Si carrier wafer coated with vacuum grease before being introduced into the etching chamber.

## Results and discussion

### Stacking Ge/Si epitaxial layers on silicon

**Figure 1:**
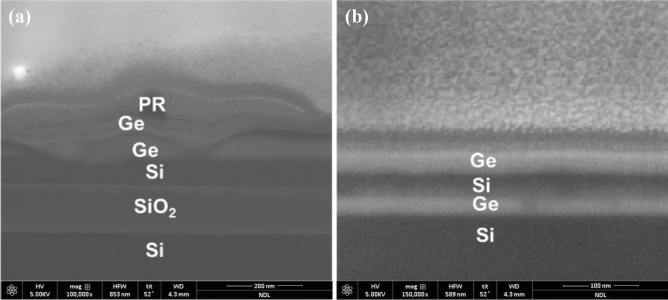
SEM images of Ge/Si multilayers. Low-temperature growth of the Si layer and ensuring that the growth process is not interrupted in the switching period between Ge and Si assist in transforming the layers from (**a**) island growth to the (**b**) 2D growth mode, despite the large Ge/Si mismatch.

**Figure 2:**
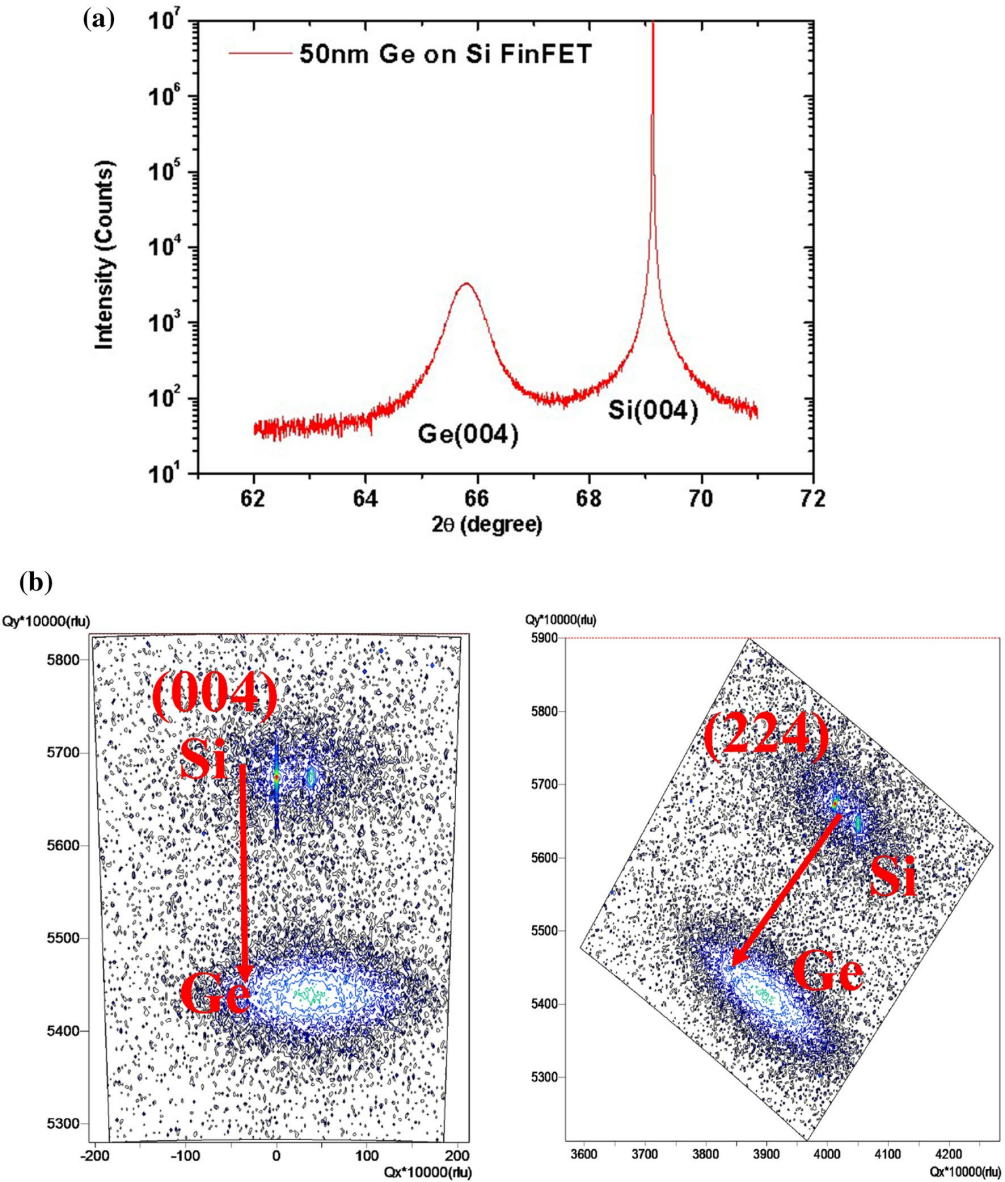
(**a**) XRD analysis and reciprocal space mapping (RSM) of the formation of a relaxed single-crystalline Ge (100) layer on the wafers and (**b**) RSM pattern of the Ge/Si multilayers grown two-dimensionally on the wafers.

Ge(40 nm)/Si(25 nm) layers were epitaxially grown on 40-nm-thick Si substrates and a buried oxide (BOX) thickness of 150 nm by LPCVD using GeH_4_ and SiH_4_ gases. SiGe is treated as sacrificial layers to build Ge stacked layers and form the multilayers of Ge/SiGe as starting materials in general. However, since the lattice mismatch of the multilayer is tiny, it is not easy to etch SiGe away from Ge selectively. Therefore, this study proposed using Si as sacrificial layers and forming multilayers of Ge/Si as starting materials. And then, using an alternative selected etching process that we proposed, we can easily remove Si away from Ge. Additionally, a large lattice mismatch between Ge and Si will prevent the epitaxial layers of Ge/Si from growing in a two-dimensional (2D) model^17,18^. To avoid island growth, the layer growth of Ge and Si was maintained at 400 and under 500 °C, respectively, and the growth process was not interrupted during the period of temperature ramping (Fig. 1). The XRD pattern of the sample is shown in Fig. 2, which indicates that the Ge layers are relaxed and flat in morphology because the Ge peak would otherwise be considerably dispersed in shape. High-resolution XRD analysis was conducted with (004) Ω–2θ scans and (224) reciprocal space mapping (RSM) to confirm the degree of strain relaxation and the lattice constant of Ge, as shown in Fig. 2. The position of the Ge peak coincides with that of ideal Ge (004) with a perpendicular lattice constant of ~ 5.66742 Å, implying that the epitaxial Ge film contains a small amount of compressive strain. The (224) RSM reveals that the 40-nm Ge layer grown on the wafer is in a nearly relaxed state.

### Dislocation removal from suspended Ge nanosheets

**Figure 3:**
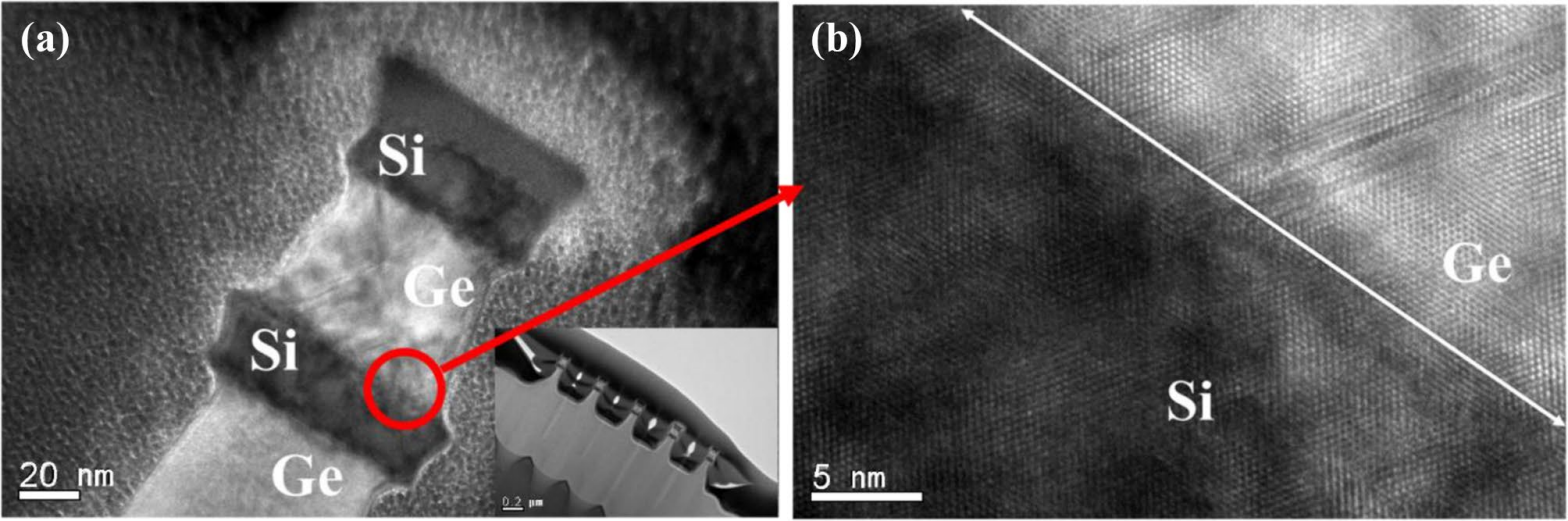
(**a**) TEM images showing misfit dislocations along the Ge/Si interface and the resulting threading dislocations across the Ge film; (**b**) enlarged micrographs with misfit dislocations at the Ge/Si interface showing the formation of a relaxed single-crystalline Ge(100) layer on the wafers.

After LPCVD-based epitaxy, the active region of the device was defined by electron-beam lithography. The active region was subsequently isolated by etching Ge/Si stacked layers using Cl_2_/HBr-related etching processes. The fin structures formed in the central area of the active region are ~ 90-nm wide (Fig. 3a). A close-up micrograph (Fig. 3b) shows Ge/SOI heterostructures and 60° misfit dislocations resulting from lattice mismatch along with the Ge/Si interface. For every misfit dislocation, two threading dislocations at the ends of the misfit exist, which must thread to the surface or form a loop through end-to-end joining. The crystal is highly strained near the dislocations. The atomic bonds surrounding the defects do not normally connect the neighbouring atoms.

**Figure 4:**
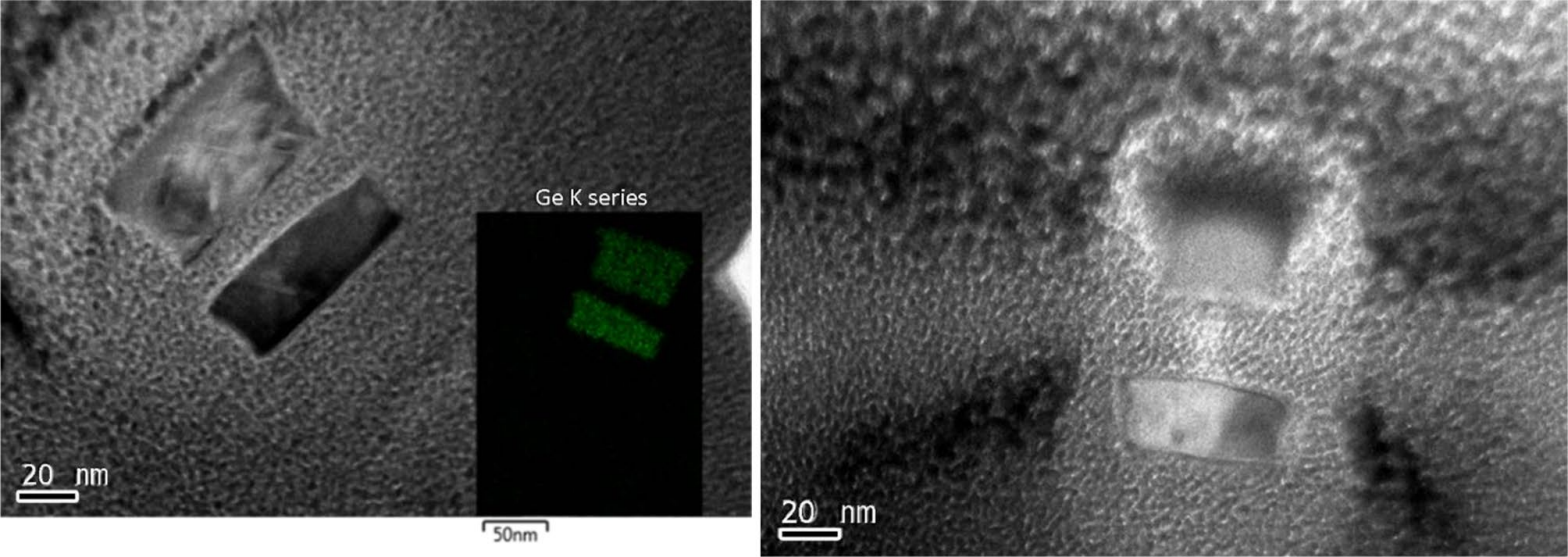
Cross-sectional TEM images of stacked Ge nanosheets (**a**) before and (**b**) after annealing. The latter show that the dislocations can be easily removed by forming gas annealing (600 °C/5 min) of the suspended sheets.

**Figure 5:**
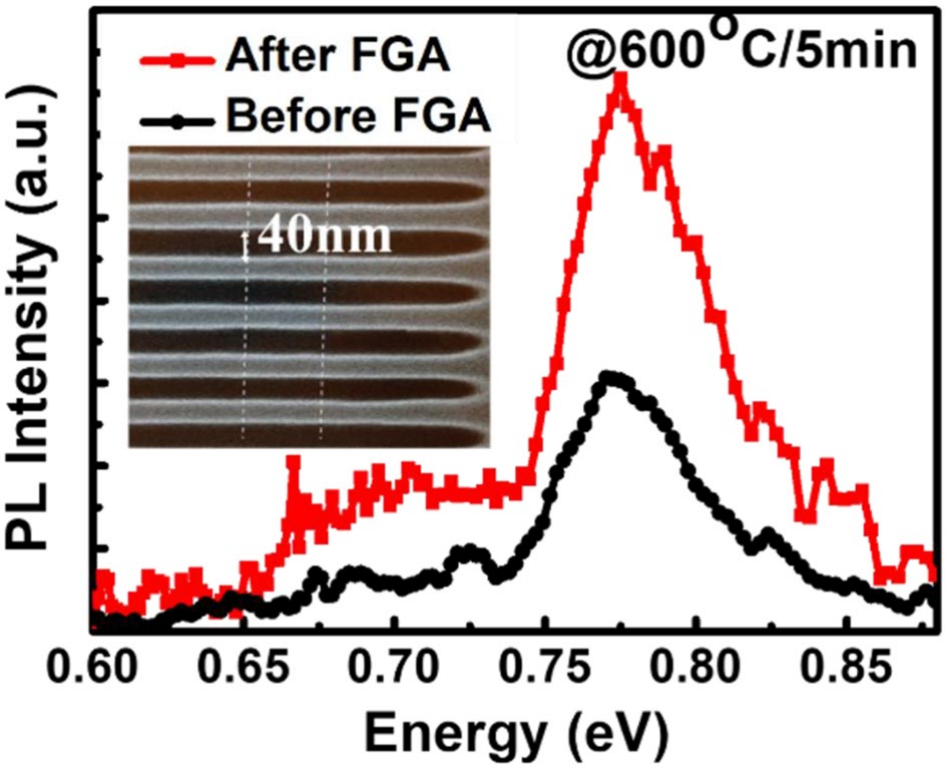
PL emission from an area with intense multiple lines of stacked Ge nanosheets. The suspended Ge nanosheets exhibit enhanced PL emission after dislocation removal by annealing.

The shape of Ge nanosheets is almost retained after etching owing to the excellent selective etching. Additionally, the dislocations in suspended Ge sheets are more easily removable than from samples with Ge layers tied to Si layers. Regarding selective etching, this study demonstrates that at a suitable temperature and with the assistance of megasonic agitation, Si layers can be readily etched away by Ge layers with good selectivity using tetramethylammonium hydroxide (TMAH) water solution, which helps retain the Ge layers. The solution must be at ~ 60 °C and employ megasonic agitation to achieve adequate.

selective etching between Ge and Si^19^. The remaining Ge sheets appear perfect. The dislocations in Ge layers are detrimental to the device performance owing to the large lattice mismatch between Ge and Si; however, the dislocations in the suspended Ge structures can be easily removed by forming gas annealing (FGA; Fig. 4a), possibly because the dislocations tend to glide more without dragging the Ge/Si interface (Fig. 4b). The reduction of dislocation density in suspended Ge was also confirmed by PL measurements (Fig. 5). Two band-edge PL peaks corresponding to recombination through the direct band-gap of Ge at 0.75 eV are observed in increasing order of wavelength. The PL peak positions are obtained as a function of excitation laser power for both samples, which shows that the PL peak energy of the Ge nanosheets agrees with the direct band-gap PL peak energy of Ge. This agreement suggests that the observed PL from the Ge nanosheets originates from direct band-gap recombination. The observed PL peak shifts towards longer wavelengths with increasing excitation laser power owing to laser-induced heating and subsequent trapping of heat within the dense array of Ge nanosheets. Additionally, the PL peak intensity shows a nearly quadratic dependence on excitation laser power and increases with increasing temperature, similar to the direct band-gap PL behaviour of bulk Ge crystals. These observations indicate that efficient direct band-gap recombination is responsible for the observed PL from the Ge nanosheets.

### Device fabrication and characterisation

**Figure 6:**
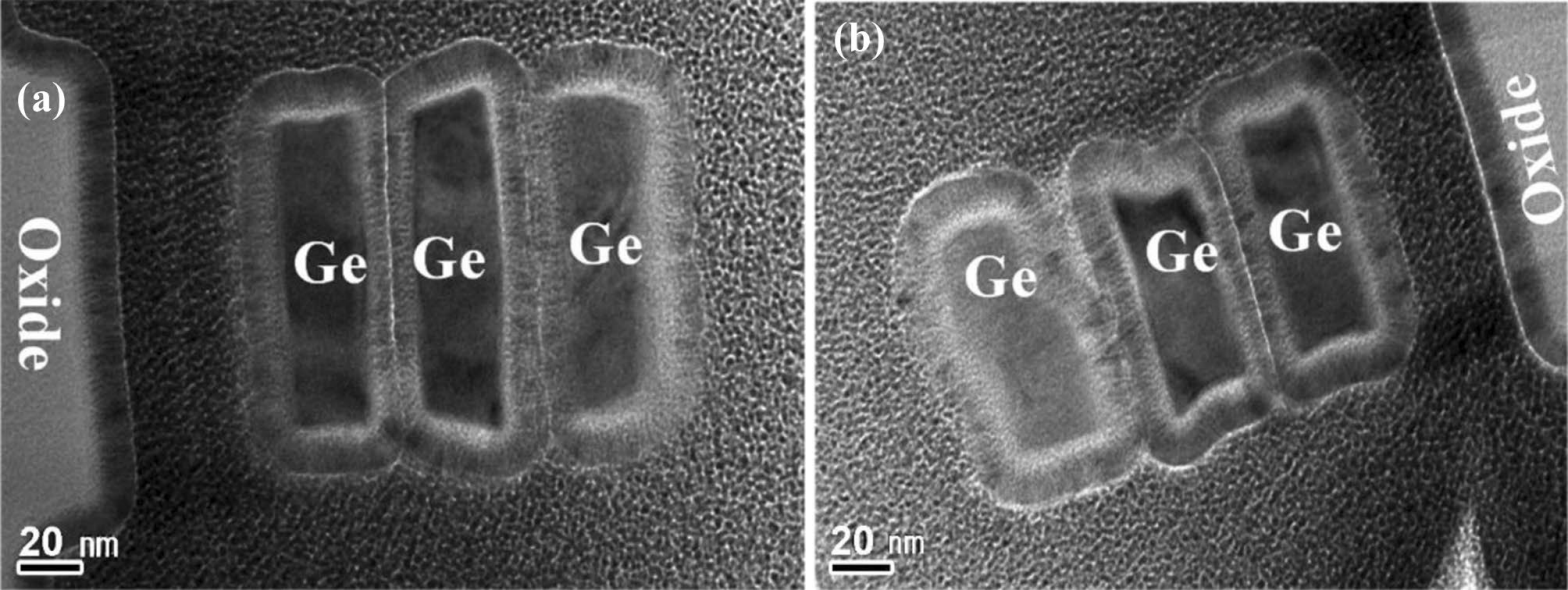
Cross-sectional TEM images of the stacked Ge nanosheets (**a**) before and (**b**) after annealing. The latter show that the dislocations can be easily removed by annealing (FGA, 600 °C/5 min) the suspended sheets.

Figure 6 shows three horizontally-stacked pure-Ge-nanosheet GAA FETs; the remaining Ge sheets appear perfect. The dislocations in the suspended Ge structures can be easily removed by FGA (600 °C/5 min), possibly because the dislocations tend to glide more without dragging the Ge/Si interface. The removal of dislocations from suspended Ge layers is easier because the line tension force (F_H_) exerted on dislocation lines at the Ge/Si interface^10^ is absent, and consequently, the net glide force (F_D_) can drive a more efficient gliding of the dislocations. A 3-nm-thick high-κ dielectric Y_2_O_3_ layer was conformally deposited around the stacked channels by atomic layer deposition (ALD) as the gate dielectric.

**Figure 7:**
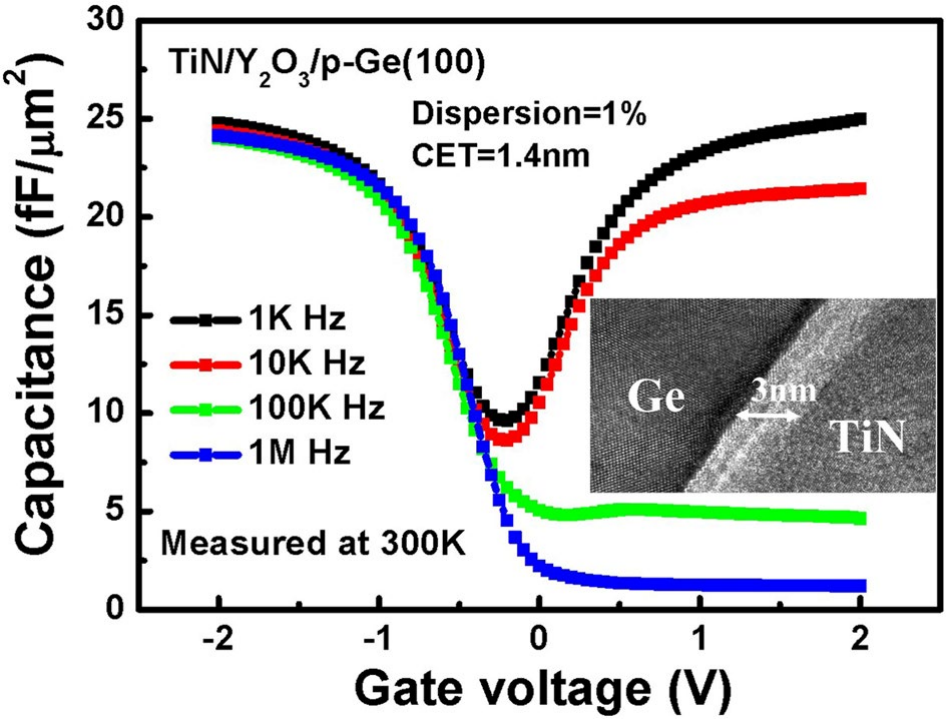
C–V characteristics of capacitors fabricated using the TiN/Y2O3 gate stack process.

The capacitance–voltage (*C-V*) curves in Fig. 7 reveal the process control of this gate stack. The *C-V* characteristics are obtained as a function of frequency for TiN/Y_2_O_3_/p-Ge structures at different cycles, and 300 K. Typical *C-V* behaviours with three distinct regions of accumulation, depletion, and inversion is observed in all the tested structures. A common feature of all the *C-V* profiles involves the stretching out that appears in the depletion region, suggesting that interfacial traps are distributed at the interface of the dielectric film and the semiconductor. The capacitance equivalent thickness (CET) is ~ 1.4 nm. Preliminary results on the TiN/Y_2_O_3_/p-Ge structures that undergo FGA reveal a decrease in the equivalent oxide thickness (EOT) due to a substantial reduction in the *C-V* profiles, indicating that ALD is a promising Y_2_O_3_ candidate for metal-oxide/Ge gate stacks.

**Figure 8:**
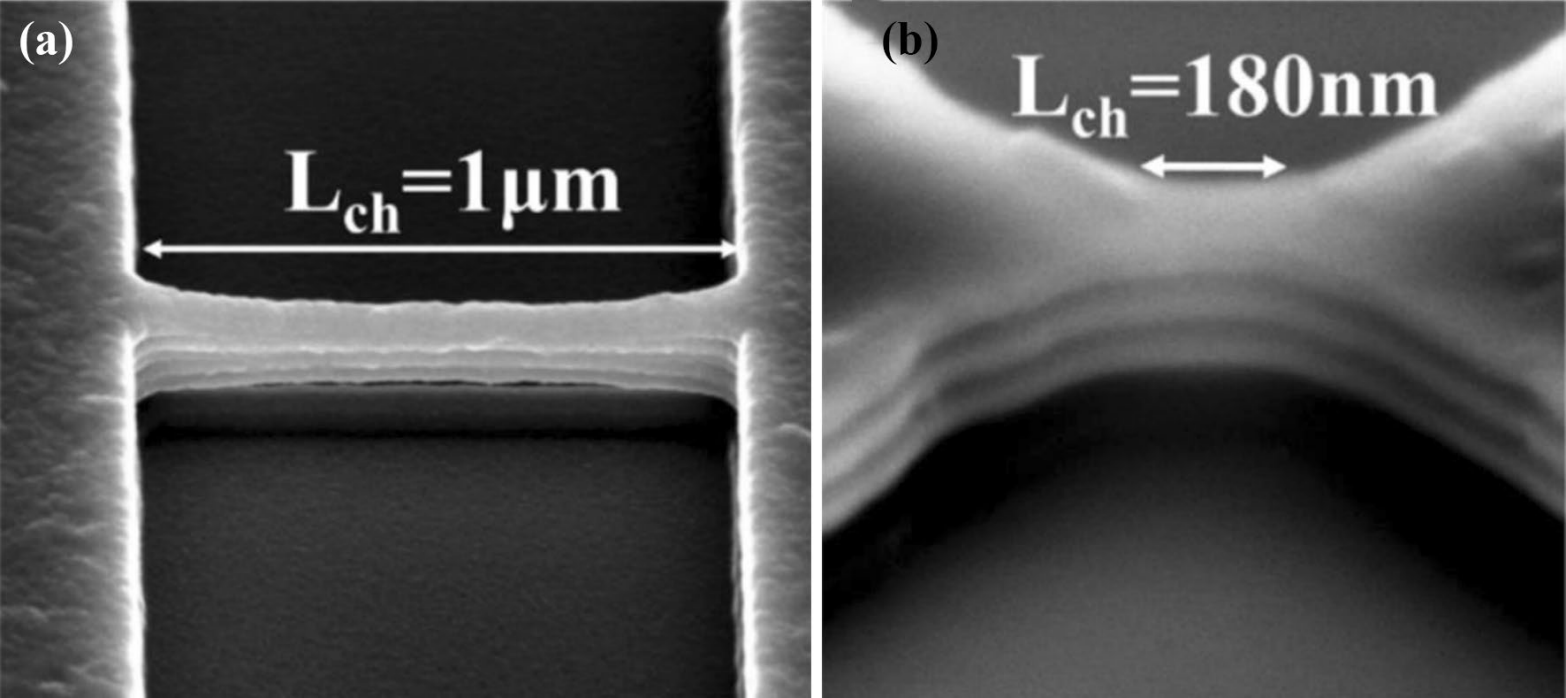
SEM tilting-view images of the stacked Ge nanosheets: (**a**) long structure of 1.0 μm and (b) short structure of 180 nm.

**Figure 9:**
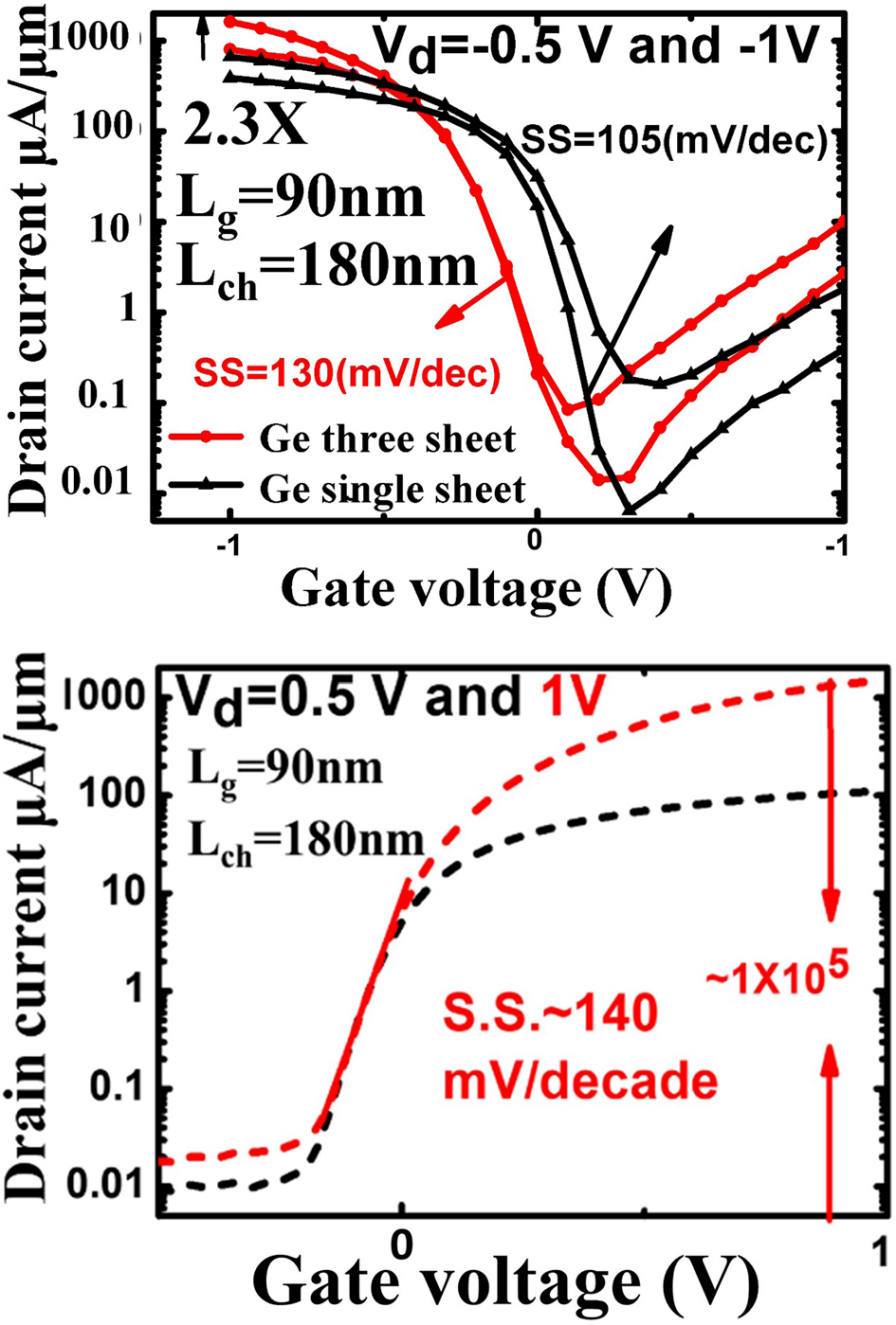
(**a**) Id—Vg curves of a three stacked Ge nanosheet and single-nanosheet P-FETs with Lch = 180 nm; the former has an enhanced ON current of ~ 2.3 times that of the latter owing to the stacked channels. (**b**) Id—Vg curves of a three stacked Ge nanosheet n-FET device.

**Figure 10:**
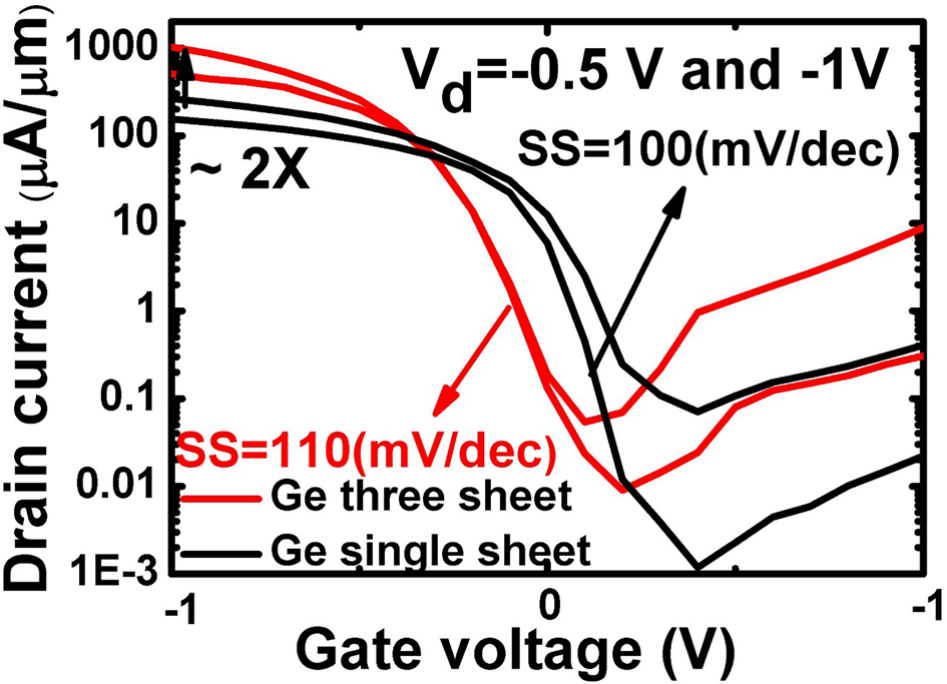
Id—Vg curves of the three stacked Ge nanosheet and single Ge nanosheet p-FETs with Lch = 1.0 µm; the former has an enhanced ON current of ~ 2.0 times that of the latter.

**Figure 11:**
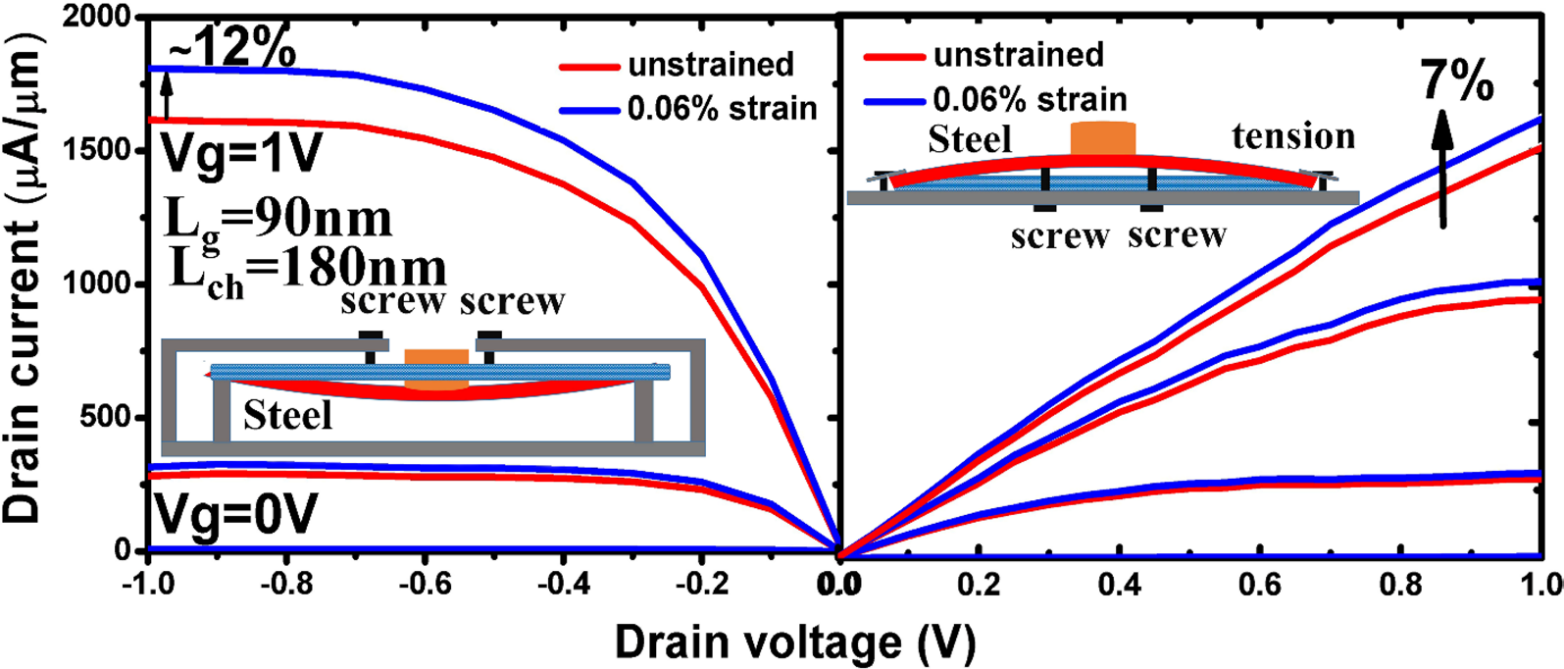
Enhancement of Id—Vg profiles of stacked Ge-nanosheet GAAFETs (Lch = 180 nm) by wafer bending. Current enhancement of 12% for p-FET and 7% for n-FET are obtained with compressive and tensile strains of 0.06%, respectively.

**Figure 12:**
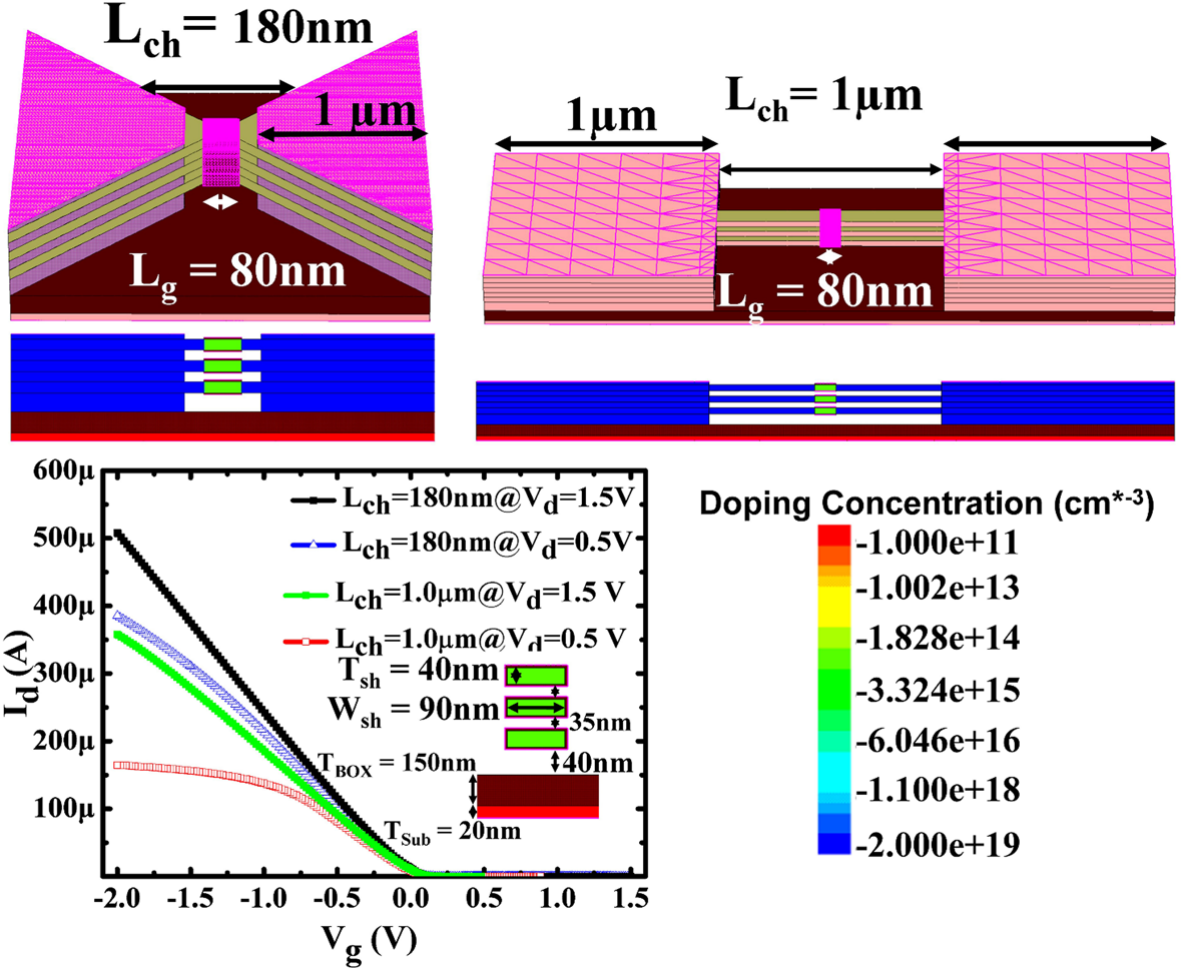
(**a**) TCAD simulation of the stacked Ge-nanosheet p-FETs with Lg = 80 nm and different Lch of 1.0 μm and 180 nm. (**b**) Simulated Id–Vg curves (linear scale) of the stacked Ge nanosheet p-FETs with Lch = 1.0 μm and 180 nm.

Stacked Ge nanosheets with lengths of 1 µm and 180 nm obtained after etching away the Si inter-layers are shown in Fig. 8. After gate formation, a three-step B implantation with doses and energies of 2 × 10^15^/cm^2^ and 30 keV, 1 × 10^15^/cm^2^ and 20 keV, and 1 × 10^15^/cm^2^ and 10 keV was performed sequentially for p-FET source/drain (S/D) uniform doping; a similar process was employed for n-FET S/D doping. Both types of doping activation were performed by two-step rapid thermal annealing: 400 °C/5 min followed by 550 °C/30 s. Figure 9 shows the drain current–gate voltage (*I*_*d*_*–V*_*g*_) characteristics of the p- and n-type Ge- nanosheet FETs; both devices are noted to exhibit decent transistor characteristics. Figure 9a shows the Id–Vg curves of a three stacked Ge-nanosheet p-FET and a single Ge-nanosheet p-FET with a short channel of 180 nm. The three-sheet device exhibits an ION current of 1650 μA/m at a gate voltage of − 1 V in Fig. 9a, which is ~ 2.3 times that of the single-sheet device. Figure 9b shows *I*_*d*_–*V*_*g*_ curves of three stacked Ge-nanosheet n-FETs. Figure 10 shows data corresponding to devices with a long channel of *L*_*ch*_ = 1.0 µm. Because of the large series resistance, all these devices exhibit smaller ION values than those of devices with the short channel *L*_*ch*_ of 180 nm, which suggests the importance of channel length for controlling the ION of a nanowire or nanosheet device. Uniaxial compressive and tensile stresses were applied to the p- and n-FETs, respectively, along the [110] channel direction by bending the sample to further enhance the ION. The applied strain was adjusted using the wafer curvature. With a strain of 0.06%, the ION of p-FETs and n-FETs can be boosted by ~ 12% and 7%, respectively (Fig. 11). The *I*_*d*_*–V*_*g*_ curves roughly correspond to the linear regime because the S/D contacts in these devices are not optimised, and the resulting serial resistance is slightly large. Finally, the Ge-nanosheet FETs were theoretically modelled by a technology computer-aided design (TCAD) simulation, as shown in *L*_*ch*_ = 1.0 µm and 180 nm. The device operation was simulated in a size-and-field regime where carrier conduction occurred on the surface of the device; the simulated dopant concentration is shown in Fig. 12a. The simulations in Fig. 12 also indicate that the long-channel device exhibits serious current degradation; the doping concentration in the S/D region (blue part in the figures) is noted to be 2 × 10^19^/cm^3^. The upper part shows their structures, and the lower part shows the carrier distribution (inset rep resents the bar scale). The currents flowing through the Ge channels in Ge-nanosheet p-FETs with *L*_*ch*_ = 1.0 µm and 180 nm are shown in Fig. 12b. The stacked Ge-nanosheet architecture is noted to provide optimal electrostatic confinement with the associated benefits of the short-channel effect, and the low levels of doping can reduce the corner effect at the threshold voltage. Compared to the bottom-up process employed for Ge-nanowire formation, the important advantages of the fabrication herein include excellent 2D Ge/Si multilayer epitaxy, novel selective Si etching over Ge using TMAH at 60 °C with megasonic agitation, and a new mechanism of dislocation removal from suspended Ge-nanosheet structures or from bulk Si substrates by annealing to meet the sub-3-nm node-related targets of the International Technology Roadmap for Semiconductors. Therefore, an entirely realisable, nearly defect-free, and relatively high-ION Ge nanosheet was developed in this study.

## Conclusions

Misfit dislocations are known to be mostly concentrated near the Ge/Si interfacial region; those generated on the heterostructure junction extend through the Ge layer and form threading dislocations. Moreover, recombination surrounds these defects, which can be easily eliminated by etching. In this study, large mismatch Ge/Si multilayers rather than Ge/GeSi multilayers were intentionally grown as the starting material for improved selective etching between Ge and Si to realise the formation of well-defined stacked Ge nanosheets. Ge/Si multilayers were grown at a low temperature to avoid island growth. The Si layers were found to be readily etched away over the Ge layers at an appropriate temperature with good selectivity using a TMAH solution. Additionally, the dislocations in suspended Ge sheets were more easily removed than from samples with Ge layers tied to Si layers. Finally, fully gated p-type and n-type transistors with three vertically-stacked Ge nanosheets as channels were developed.

## Abbreviations

GAAFETs: Gate-all-around field-effect transistors
SOI: Silicon-on-insulator
SC1&SC2: Standard cleaning 1 & 2
LPCVD: Low-pressure chemical vapour deposition
TEM: Transmission electron microscopy
PL: Photoluminescence
XRD: X-ray diffraction
TCP: Transfer-coupled plasma
BOX: Buried oxide
RSM: Reciprocal space mapping
TMAH: Tetramethylammonium hydroxide
FGA: Forming gas annealing
ALD: Atomic layer deposition
F_D_: Net glide force
C–V: Capacitance–voltage curve
CET: Capacitance equivalent thickness
S/D: Source/drain
I_d_/V_g_: Drain current—gate voltage
TCAD: Technology computer-aided design
EOT: Equivalent oxide thickness
